# Dietary type 2 resistant starch improves systemic inflammation and intestinal permeability by modulating microbiota and metabolites in aged mice on high-fat diet

**DOI:** 10.18632/aging.103187

**Published:** 2020-05-25

**Authors:** Yawen Zhang, Luyi Chen, Mengjia Hu, John J. Kim, Renbin Lin, Jilei Xu, Lina Fan, Yadong Qi, Lan Wang, Weili Liu, Yanyong Deng, Jianmin Si, Shujie Chen

**Affiliations:** 1 Department of Gastroenterology, Sir Run Run Shaw Hospital, School of Medicine, Zhejiang University, Hangzhou 310016, Zhejiang Province, China; 2 Institute of Gastroenterology, Zhejiang University, Hangzhou 310016, Zhejiang Province, China; 3 Division of Gastroenterology, Loma Linda University Health, Loma Linda, CA 92354, USA

**Keywords:** resistant starch, high-fat diet, aging, microbiome, inflammation

## Abstract

Type 2 resistant starch (RS2) is a fermentable dietary fiber conferring health benefits. We investigated the effects of RS2 on host, gut microbiota, and metabolites in aged mice on high-fat diet. In eighteen-month old mice randomly assigned to control, high-fat (HF), or high-fat+20% RS2 (HFRS) diet for 16 weeks, RS2 reversed the weight gain and hepatic steatosis induced by high-fat diet. Serum and fecal LPS, colonic IL-2 and hepatic IL-4 mRNA expressions decreased while colonic mucin 2 mRNA and protein expressions increased in the HFRS compared to the HF and the control group. 16s rRNA sequencing of fecal microbial DNA demonstrated that RS2 decreased the abundance of pathogen taxa associated with obesity, inflammation, and aging including *Desulfovibrio* (*Proteobacteria* phylum), *Ruminiclostridium 9*, *Lachnoclostridium*, *Helicobacteria, Oscillibacter*, *Alistipes*, *Peptococcus,* and *Rikenella.* Additionally, RS2 increased the colonic butyric acid by 2.6-fold while decreasing the isobutyric and isovaleric acid levels by half compared to the HF group. Functional analyses based on Clusters of Orthologous Groups showed that RS2 increased carbohydrate while decreasing amino acid metabolism. These findings demonstrate that RS2 can reverse weight gain, hepatic steatosis, inflammation, and increased intestinal permeability in aged mice on high-fat diet mediated by changes in gut microbiome and metabolites.

## INTRODUCTION

Popularization of western dietary habits has led to higher consumption of energy-dense food, rich in fat, world-wide [1]. Consumption of high-fat diet is associated with aging and disease processes including metabolic syndrome, cardiovascular disease, and colon cancer [2–4]. Furthermore, the ill-effects of high-fat diet are more pronounced in older age by increasing the susceptibility to metabolic syndrome [5], neuroinflammation [6], and hepatic fibrosis [7]. The prevalence of diet-related obesity among middle-aged adults and elderly is also higher compared to younger adults [8]. There is growing interest in interventions aiming at prevention of ill-effects of high-fat diet in the middle-aged and elderly population.

Resistant starch (RS), a type of dietary fiber, has been extensively studied for the past few decades conferring broad range of health benefits including improvement in glycemic control, weight loss in obesity, and reduction in the risk of colon cancer [9]. Type 2 resistant starch (RS2) as raw granules has been widely evaluated in animal and human studies [10, 11]. FDA has approved Hi-maize resistant starch, a commercial RS2 supplementation produced from naturally modified high amylose corn, for use in patients with type 2 diabetes. Furthermore, recent studies demonstrated that RS2 may improve host responses and brain functions in aged animals [12–14]. However, whether RS2 can improve host health specifically in middle-aged and elderly population on high-fat diet is unclear and the underlying mechanism is largely unknown.

In the gastrointestinal tract, both high-fat diet and aging lead to gut microbiota dysbiosis [15, 16], and emerging studies demonstrate that altering gut dysbiosis may potentially mitigate the harmful effects of high-fat diet and aging [17, 18]. As a fiber that can be fermented by colonic commensal bacteria with production of short-chain fatty acids (SCFA), we hypothesize that RS2 may provide therapeutic effects of altering microbiota. The aim of the study was to investigate the effects of RS2-mediated modulation of microbial community structure, composition, and metabolic functions on weight gain, systemic inflammation, and intestinal permeability in aged mice fed with high-fat diet.

## RESULTS

Prior to the completion of the experiment at 16 weeks, one mouse in the control group died at 14 weeks due to dramatic weight loss with unexplored reasons. After completing the dietary intervention at 16 weeks, one mouse in HF (high-fat diet) group developed gastrointestinal bleeding possibly caused by a liver mass during sacrifice. Therefore, stool collection for microbiota and metabolites analysis was not possible.

### Effects of RS2 treatment on body weight, food intake, colon, and liver histopathology in aged mice on high-fat diet

**Figure 1:**
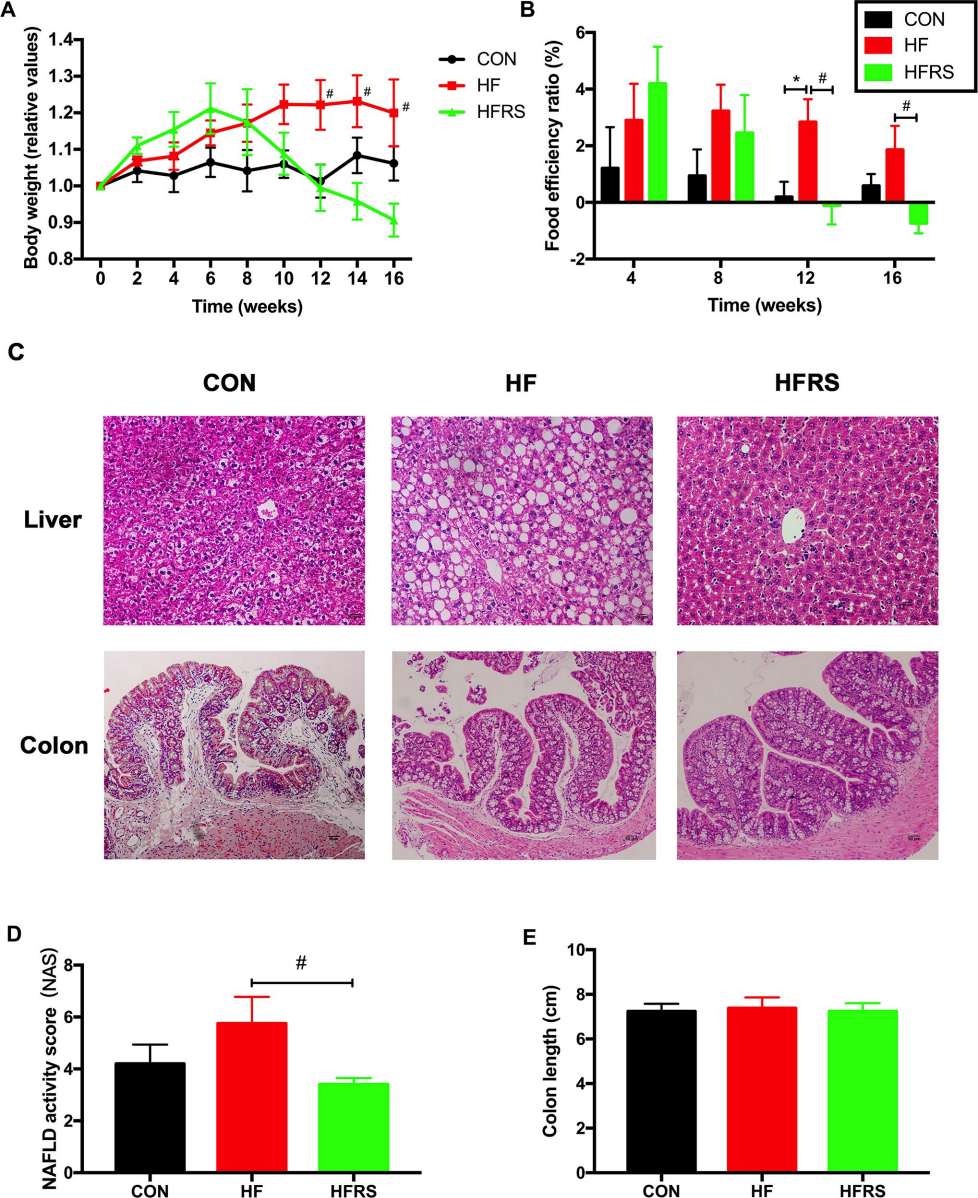
**RS2 reduced body weight gain and liver NAFLD activity score in aged mice on high-fat diet.** (**A**) Effects of diets on relative values of body weight every two weeks. (**B**) Monthly food efficiency ratio among the three groups. (**C**) Liver and colon histology on H&E staining slides (magnification, 200X) among the three groups. (**D**) Comparison of NAFLD activity scores calculated by the average score of three fields in each H&E staining slides (magnification, 400X). (**E**) Comparison of colon length among the three groups. n=5 or 6/group. Data are expressed as mean+SE. Differences were compared by one-way ANOVA among the three groups with Tukey’s multiple comparison posttests between the two groups. * p<0.05 compared with CON group, # p<0.05 compared with HFRS group. CON, control group; HF, high-fat diet group; HFRS, high-fat diet+20%RS2 group.

At 12 weeks, a trend towards weight gain was observed in the HF compared to the control group (p=0.06). However, the weight gain was counteracted in the HFRS (high-fat diet+20% RS2) group at 12 weeks and beyond (p<0.05, Figure 1A). Furthermore, food efficiency ratio was higher in the HF compared to the control group at 12 weeks, and lower in the HFRS compared to the control group at 12 and 16 weeks (p<0.05, Figure 1B). Liver histopathology demonstrated higher non-alcoholic fatty liver disease (NAFLD) activity in the HF group compared to the HFRS group (p<0.05, Figure 1C, 1D). No differences in colon histopathology scores and colon length were found among the three groups (Figure 1C, 1E).

### Effects of RS2 treatment on gut permeability and inflammation in aged mice on high-fat diet

**Figure 2:**
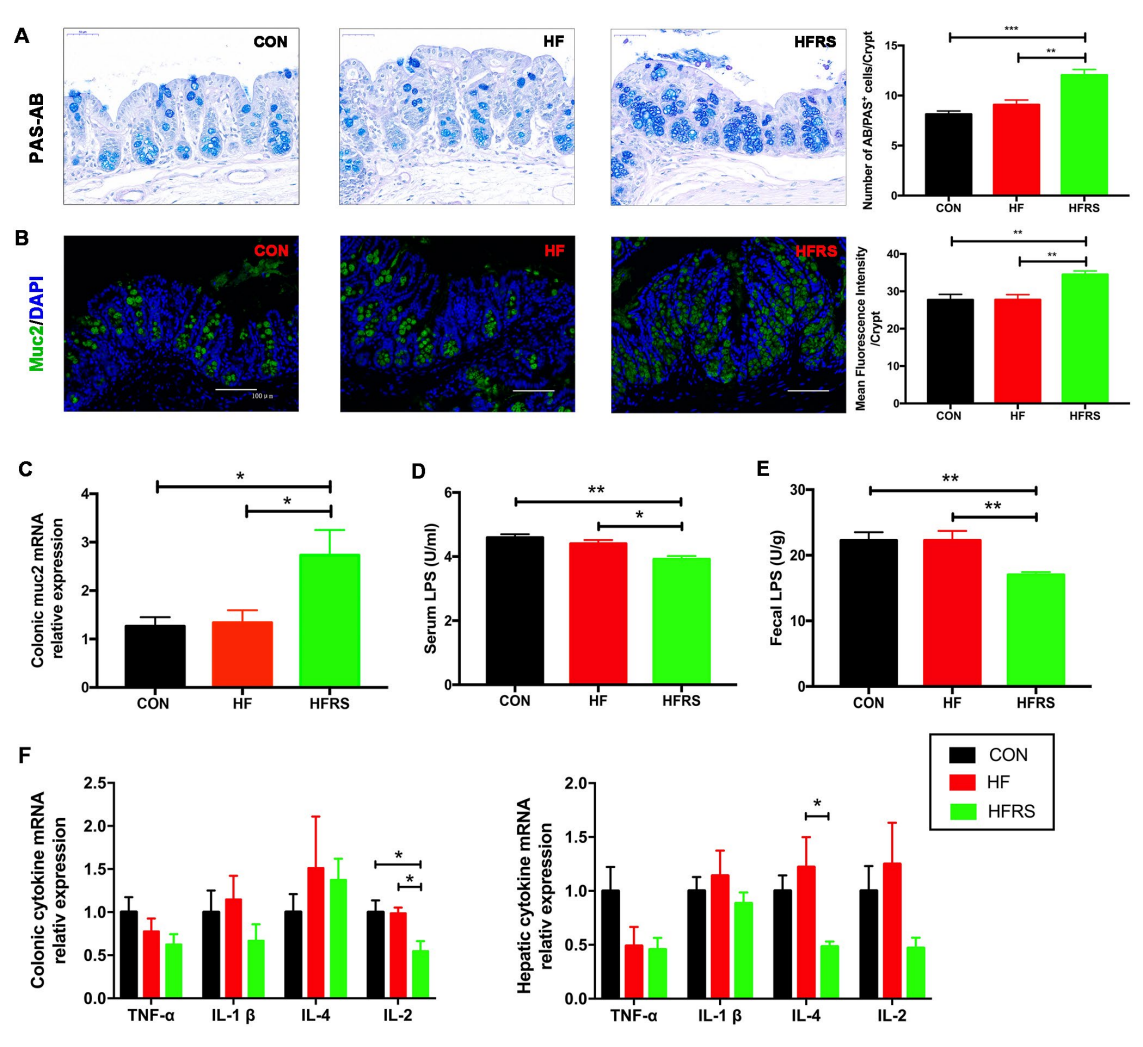
**RS2 reduced intestinal permeability and inflammation in blood, colon, and liver in aged mice on high-fat diet.** (**A**) Representative images of PAS-AB staining in colon tissues from three groups (400x magnification, scale bar =50 μm) and the quantification of PAS-AB positive cells per crypt for every mouse in each group. (**B**) Immunofluorescence staining of MUC2 (green) with nuclear counterstaining (blue) in colon tissue from the three groups (200x magnification, scale bar=100 μm) and quantification analysis of mean fluorescence intensity for every mouse in each group. (**C**) Effects of diet on colonic MUC2 mRNA expression assessed by quantitative real-time PCR. (**D**) Comparison of serum LPS levels assessed by ELISA among the three groups. (**E**) Comparison of fecal LPS levels assessed by ELISA among the three groups. (**F**) Effects of diets on colonic and hepatic inflammatory cytokines mRNA expression assessed by quantitative real-time PCR. Multiple comparisons of colonic TNF-α levels (p=0.22); multiple comparisons of hepatic TNF-α levels (p=0.07). n=5 or 6/group. Data are expressed as mean+SE. Differences were compared by one-way ANOVA among the three groups with Tukey’s multiple comparison posttests between two groups. * p<0.05, ** p<0.01, *** p<0.001. MUC2, mucin2; CON, control group; HF, high-fat diet group; HFRS, high-fat diet+20%RS2 group.

Mucin expression levels in colon, lipopolysaccharide (LPS) concentration in blood and stool, and inflammatory cytokines mRNA expression levels in colon and liver of each group were examined. RS2 supplementation increased the colonic MUC2 expression of protein (Figure 2A, 2B) and mRNA (2.73+0.52 in HFRS vs. 1.34+0.26 in HF group, p=0.03; 2.73+0.52 in HFRS vs. 1.27+0.19 in control, p=0.02, Figure 2C) in the HFRS group compared to the HF and the control groups. Furthermore, RS2 supplementation reduced LPS levels in serum (3.92+0.10 in HFRS vs 4.40 + 0.11 in HF, p=0.02; 3.92+0.10 in HFRS vs 4.59+0.10 in control, p=0.001, Figure 2D) and stool (17.01+0.44 in HFRS vs 22.25+1.47 in HF, p=0.008; 17.01+0.44 in HFRS vs 22.27+1.26 in control, p=0.008, Figure 2E) of the HFRS compared to the HF and the control groups.

Among inflammatory cytokines, HFRS group had lower colonic interleukin-2 (IL-2) expression compared to the HF and the control groups (0.55+0.12 in HFRS vs. 0.99+0.07 in HF, p=0.04; 0.55+0.12 in HFRS vs. 1.00+0.14 in control, p=0.03, Figure 2F). HFRS group also had lower hepatic interleukin-4 (IL-4) expression compared to the HF group (0.48+0.05 in HFRS vs. 1.22+0.28 in HF, p=0.02, Figure 2F). A trend towards reduced hepatic tumor necrosis factor (TNF-α) expression in the HFRS (0.46+0.11 in HFRS vs. 1.0+0.22 in control, p=0.08) and a non-significant reduction of hepatic TNF-α in the HF (0.49+0.18 in HF vs. 1.0+0.22 in control, p=0.15) compared to the control group were observed. No differences in other inflammatory cytokine of expression levels were found including colonic TNF-α, colonic and hepatic interleukin-1(IL-1β), colonic IL-4, and hepatic IL-2 among the three groups (Figure 2F).

### Effects of RS2 treatment on gut microbial diversity and composition in aged mice on high-fat diet

**Figure 3:**
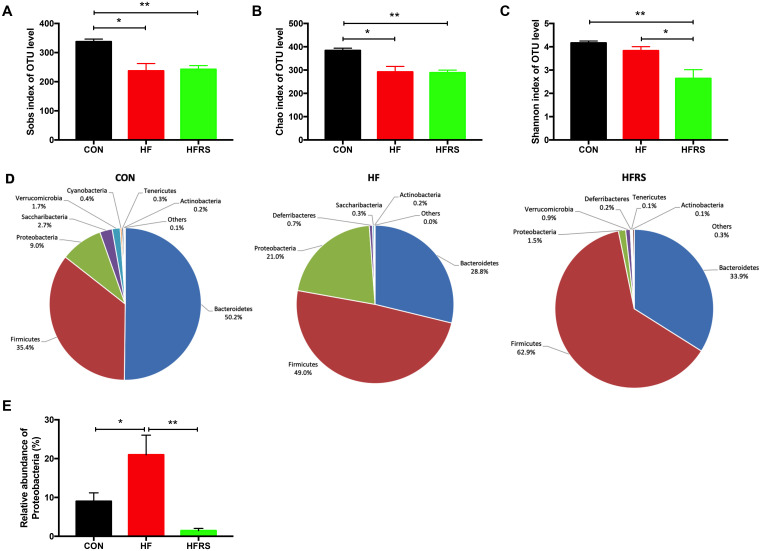
**High-fat diet and RS2 supplemented with high-fat diet altered the microbial diversity and community at the phylum level in aged mice.** (**A**–**C**) Alteration of α-diversity induced by HF diet and HFRS diet. (**A**) Sobs index and (**B**) Chao1 index represent richness of community, (**C**) Shannon index represents diversity of community. (**D**) Pie chart of phyla alteration caused by HF diet and HFRS diet. (**E**) Relative abundance of *Proteobacteria* among the three groups. n=4 to 6/group. Data are expressed as mean+SE. Differences were compared by one-way ANOVA among the three groups with Tukey’s multiple comparison posttests between the two groups. * p<0.05, ** p<0.01. CON, control group; HF, high-fat diet group; HFRS, high-fat diet+20%RS2 group.

To evaluate the impact of dietary interventions on gut microbiota, 16s rRNA sequencing of the mice fecal DNA were performed at 16 weeks. The microbiota analysis showed that high-fat diet reduced the richness of microbiota (Sobs and Chao1 index in OTU level, Figure 3A, 3B) but not diversity (Shannon index in OTU level, Figure 3C). No difference in richness was observed between the HFRS and the HF groups. However, HFRS demonstrated reduced diversity compared to the HF and the control groups (P<0.05, Figure 3C).

**Figure 4:**
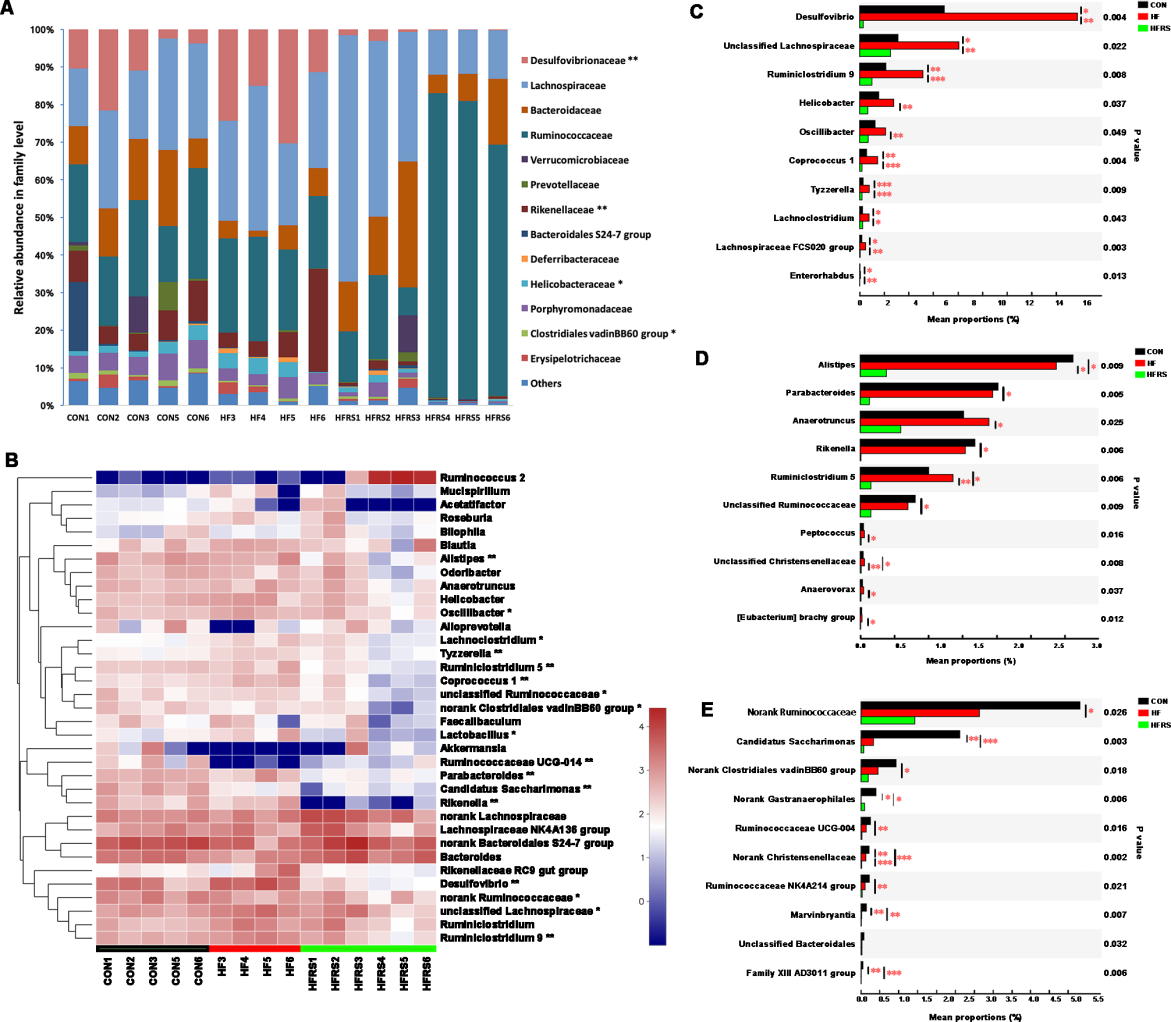
**High-fat diet and RS2 supplemented with high-fat diet altered the microbial community at the family and genus levels in aged mice.** (**A**) Relative abundance of gut microbiota in family level (those with abundance >1% are presented) among the three groups. (**B**) Top 35 taxa with the highest abundance at the genus level among the three groups. Colors were expressed by lg calculation. (**C**) Generic taxa with higher mean proportions in the HF and lower mean proportions in the HFRS groups compared to the CON group by further posttest comparisons. (**D**) Generic taxa with lower mean proportions in the HFRS compared to the HF and the CON groups by further posttest comparisons. (**E**) Generic taxa with lower mean proportions in the HF and the HFRS compared to the CON group by further posttests comparisons. N=4 to 6 per group. Differences were compared by Kruskal–Wallis H test with Dunn’s multiple posttest comparisons between the two groups. * p<0.05, ** p<0.01. CON, control group; HF, high-fat diet group; HFRS, high-fat diet+20%RS2 group.

Abundance of bacteria at the phylum level by the groups were analyzed (Figure 3D). Higher relative abundance of *Proteobacteria* was observed in the HF compared to the control group (21.00% vs. 9.01%, P<0.05, Figure 3E). However, HFRS had lower relative abundance of *Proteobacteria* compared to both the HF (1.47% vs. 21.00%, P<0.01) and the control (1.47% vs. 9.01%, P<0.01) groups (Figure 3E). Further analysis at the family and genus levels demonstrated that the abundance of bacteria including *Bacteroidetes* (i.*e. Rikenellaceae*, *Parabacteroides*, *Alistipes*), *Firmicutes* (i.e. *Clostridiales vadinBB60 group*, *Ruminiclostridium 9*, *Ruminiclostridium 5*, *Oscillibacter*, *Coprococcus 1*, *Lactobacillus*, *Tyzzerella*, *Lachnoclostridium*), and *Proteobacteria* (e.g. *Desulfovibrionaceae, Helicobacteraceae*) species were different among the three groups (p<0.05, Figure 4A, 4B).

Two group comparisons showed that the HFRS group had lower relative abundance of certain bacteria species (i.e. *Desulfovibrio*, *Ruminiclostridium 9*, *Helicobacter*, *Oscillibacter*, *Coprococcus 1*, *Tyzzerella*, *Lachnoclostridium*, *Lachnospiraceae FCS020 grou*p, *Enterorhabdus* and *unclassified Lachnospiraceae)* compared to the HF group; HF group had higher relative abundance of the same species compared to the control group (Figure 4C). Furthermore, HFRS group demonstrated lower relative abundance of some taxa (i.e. *Alistipes*, *Parabacteroides*, *Anaerotruncus*, *Rikenell*a, *Ruminiclostridium 5*, *Peptococcus*, and *Anaerovorax*) compared to the HF and the control groups (Figure 4D). Finally, the relative abundance of *Candidatus Saccharimonas*, *Ruminococcaceae UCG-004*, *Ruminococcaceae NK4A214 group*, *Marvinbryantia*, and *Family XIII AD3011* species were lower in the HF and the HFRS compared to the control group (Figure 4E).

### Metabolic effects of RS treatment in aged mice on high-fat diet

**Figure 5:**
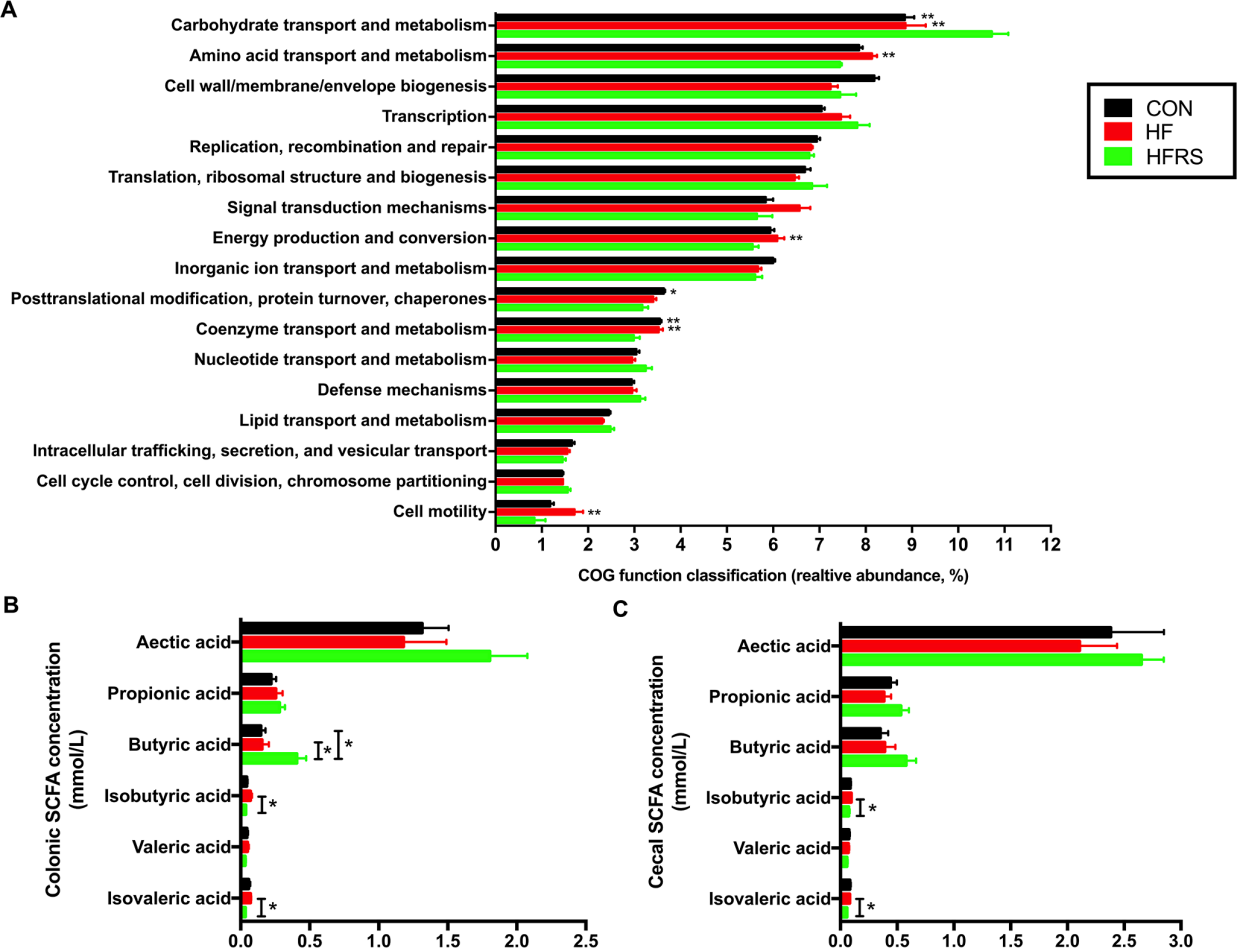
**High-fat diet and RS2 supplemented with high-fat diet altered the microbial metabolism in aged mice.** (**A**) Functional prediction analyses based on the occurrence of clusters of orthologous groups (COGs) of proteins in microbiota among the three groups (those with abundance >1% are presented). (**B**) Colon short-chain fatty acid levels regulated by HF and HFRS diets. (**C**) Cecal short chain fatty acid levels regulated by HF and HFRS diets. N=4 to 6 per group. Data are expressed as mean + SE. Differences were compared by one-way ANOVA among the three groups with Tukey’s multiple comparison posttests between two groups or Kruskal–Wallis H test with Dunn’s multiple comparisons posttests between two groups. * p<0.05, ** p<0.01 compared with HFRS or CON. CON, control group; HF, high-fat diet group; HFRS, high-fat diet+20%RS2 group; SCFA, short-chain fatty acid.

Functional analyses based on the occurrence of clusters of orthologous groups (COGs) of proteins showed that HFRS group had higher carbohydrate, but lower amino acid metabolism compare to the HF and the control groups. Furthermore, HFRS group showed lower energy production and conversion, coenzyme transport and metabolism, and cell motility compared to the HF and the control groups. (Figure 5A).

Evaluation of SCFA showed that the HFRS group had higher levels of butyric acid in colon compared to the HF and the control groups (p<0.05, Figure 5B). Meanwhile, HFRS group had lower concentrations of isobutyric and isovaleric acids in the cecum and colon compared to the HF group (p<0.05, Figure 5B, 5C).

## DISCUSSION

We evaluated the effects of RS2 administration on inflammation and intestinal permeability in aged mouse model on high-fat diet. As expected, aged mice on high-fat diet increased body weight and developed histologic liver injury. RS2 reversed the high-fat diet induced liver injury, enhanced gut barrier function by increasing mucin expression, and exerted anti-inflammatory effects by reducing systemic endotoxemia and pro-inflammatory cytokines. Furthermore, gut dysbiosis caused by high-fat diet was altered by RS2 supplementation leading to changes in SCFA production and a shift from amino acid to carbohydrate metabolism.

Our results demonstrating the protective effect of RS2 on weight gain caused by high-fat diet is consistent with findings in previous studies [19, 20]. Although some studies failed to demonstrate reduction of total body weight [14, 21, 22], the relative weight of cecum and gastrointestinal tract increased with RS2 administration [14]. In our study, the body weight was measured in the afternoon to minimize the contribution of fecal weight in nocturnally feeding mice [23]. Furthermore, our study protocol utilized a relatively high concentration of RS2 (20%) and long duration of intervention (16 weeks) to clearly evaluate the anti-obesity effects of RS2 [20, 24]. Previous studies have also indicated that RS at lower doses may have a dose-dependent effect on reduction of adiposity in obese rats [20]. However, excessively high dose of RS (35%) may increase anxiety-like behavior in mice [24]. The concentration of RS2 was selected based on previously described methods, while minimizing the risk of adverse events in our aged mouse model [12, 21, 24]. The concomitant increase in body weight and food efficacy ratio in the HF compared to the HFRS group only after 12 weeks, suggested a delay in therapeutic effects of RS2. Our results, as well as other studies, demonstrated that reduction in body weight required >12 weeks of RS2 intervention, possibly related to the lag time required for preceding changes in gut microbiome [21, 22, 25]. In addition to weight control, our results demonstrated RS2 reduced hepatic steatosis induced by high-fat diet. Lower NAFLD activity scores in the HFRS group, specifically minimal ballooning of hepatocytes on histology, further confirmed the anti-adiposity effects of RS2. Chronic low-grade inflammation characterized by increased inflammatory cytokines are important in the pathogenesis of metabolic syndrome and cardiovascular disease in middle-age and elderly population [26, 27]. Remarkably, RS2 displayed systemic and organ-specific anti-inflammatory action by lowering serum and fecal LPS levels in our study. Reduction of fecal LPS production provides protection against mucosal injury by limiting the release of common inflammatory cytokines triggered by LPS/TLR4 signaling [28]. Furthermore, increased mucin production by RS2 supplementation enhances intestinal barrier function and prevent endotoxemia that are associated with systemic and distant organ (e.g. liver) inflammation [26, 27]. The protective effects of RS2 were supported by lower colonic and hepatic cytokine levels (IL-2 and IL-4) in aged mice in the HFRS compared to the HF group in our study. However contrary to studies in young mouse model [22, 29], a trend towards reduced hepatic TNF-α level in the HFRS group (p value=0.08) and non-significant reduction in hepatic TNF- α level in the HF group (p value=0.15) compared to the control group were observed. Activated natural killer (NK) and T cells are major producers of TNF-α, and a prior study demonstrated that BMI was associated with NK cells apoptosis in the elderly, but not in the young cohort [30]. Endogenous inflammation cytokines can also cause functional inactivation and apoptosis of NK cells and T cells under certain circumstances like aging [31, 32]. Although interpretation of results without statistical significance is uncertain, the inactivation and apoptosis of immune cells induced by diet unique to aged mice may be present.

Gut microbiota composition and metabolites were altered by dietary interventions in our study. Although RS2 supplementation did not improve the overall *α*-diversity in aged mice on high-fat diet, *Proteobacteria,* a major phylum that include LPS-producing species, was decreased with RS2 supplementation. Previous studies demonstrated that increase of LPS-producing pathogens was associated with high-fat diet [15]. Furthermore, the increase of LPS-producing bacteria *Desulfovibrio,* responsible for the production of hydrogen sulfide (H_2_S) associated with high-fat diet, was reduced with RS2 supplementation. H_2_S promotes inflammation by impairing mitochondrial respiration in colonocytes and butyrate oxidation that provides cell energy [33]. In addition, the high abundance of *Oscillibacter* species associated with obesity and impaired gut permeability was reduced with RS2 supplementation [34]. Finally, RS2 altered the relative abundance of several other taxa including *Ruminiclostridum 9,* *Lachnoclostridium*, *Helicobacteria*, and *Tyzzerella* that are increased in obesity resulting from high-fat diet [34, 35]. Although the identification and characterization of obesogenic microbiota are incomplete, our findings of RS2 reversing the increased abundance of known species in high-fat diet to the level of the control group indicate that gut microbial dysbiosis in aged mice on high-fat diet could be corrected. Our study demonstrated that higher abundance of several other pro-inflammatory taxa (*Rikenella*, *Parabacteroides*, *Peptococcus* and *Marvinbryantia*) and age-related taxa (*Alistipes*) observed in both the control and HF groups were also reduced by RS2 supplementation. *Alistipes* is the most overrepresented taxon in aged mice [36]. *Rikenella* and *Parabacteroides* are associated with inflammatory bowel disease [37, 38], while abundance of *Peptococcus* and *Marvinbryantia* are associated with intestinal inflammation in stress models [39].

As a dietary fiber, RS2 also showed prebiotic effects by enriching butyrate-producing bacteria (e.g. *Ruminococcaceae* and *Lachnospiraceae*) in our study as well as others [40, 41]. Interestingly, we observed two distinguishing bacteria profiles in the HFRS group characterized by increased abundance of *Ruminococcaceae* (N=3) or *Lachnospiraceae* (N=3*)*. Although the microbial profiles appeared different, the metabolites and therapeutic effects of RS2 in the two groups were similar (data not showed). HFRS group demonstrated the lowest relative abundance of *Lactobacillus* genus among three groups in our study. Although a variety of probiotics have demonstrated abundance of *Lactobacillus* (i.e. *Lactobacillus GG*), a recent study showed that *Lactobacillus reuteri*, a commensal *Lactobacillus* strain, drove autoimmunity in a Toll-like receptor 7-dependent mouse model of lupus [42]. In the same study, RS supplementation suppressed the abundance and translocation of *Lactobacillus* *reuteri* by increased production of SCFA in line with our findings.

The COG function classification prediction analysis in our study showed age-related microbial changes characterized by decreased saccharolytic and increased proteolytic bacteria consistent with previous studies [43]. RS2 supplementation promoted saccharolytic fermentation shown by increased levels of fecal butyrate while suppressing proteolytic fermentation evident by decreased fecal isobutyrate and isovalerate levels. Butyrate is a key metabolite in intestinal homeostasis by modulating immune response through inhibiting the release of pro-inflammatory cytokines [44] and enhancing intestinal barrier function by upregulating the expression of mucin 2. On the contrary, high levels of isobutyrate and isovalerate are associated with hepatic steatosis, metabolic syndrome, and cardiovascular disease [45]. Although unclear significance, the COG function classification analysis in the study also showed that RS2 reduced energy production/conversion, coenzyme transport/metabolism, and cell motility of bacteria.

To our knowledge, the current study is the first to show protective effect of RS2 supplementation against hepatic steatosis and weight gain in an aged animal model on high-fat diet. Our study provides preliminary mechanistic evidence for clinical application of resistant starch in middle-aged and elderly patients with cardiovascular comorbidities. Our study has limitations. First, evaluating different concentrations of RS2 may have provided a more robust evidence for the therapeutic effect on the study endpoints. Furthermore, the 16s rRNA sequencing used in our study allowed evaluation of changes in gut microbiota only at the genus level. Whole genomic analysis of gut microbiota would have provided characterization at the species level with associated pro- or anti-inflammatory properties. In addition, delineating detailed mechanism of microbial changes and host response to RS2 were beyond the scope of the study. Further studies of RS2 evaluating whole genomic analysis in microbiome to examine the effects on specific bacteria strains and focusing on detailed mechanism of the microbiota-host interaction will be invaluable. For example, studying the direct effect of SCFA by using a SCFA receptor knockout mice (GPR41^-/-^ and GPR43^-/-^mice) may better clarify the involved mechanisms.

In conclusion, RS2 supplementation reversed weight gain, hepatic steatosis, inflammation, and increased intestinal permeability in aged mice on high-fat diet. Furthermore, the therapeutic effects of RS2 supplementation were associated with altered microbiome and functional metabolites. Future clinical studies evaluating RS2 supplementation in patients with elevated risk of cardiovascular disease are needed.

## MATERIALS AND METHODS

### Animals

Eighteen-month-old female C57BL/6 mice were obtained from Slac Laboratory Animal Co. Ltd. (Shanghai, China) and housed at Sir Run Run Shaw Hospital animal facility in a pathogen-free level room. Two or four mice were cohoused in a transparent plastic feeding cage. The room temperature was maintained at 20-22°C with 12-h light/dark cycle. Mice were provided with food and water ad libitum.

### Experimental design

Mice were randomly assigned to three groups (N=6 per group) based on different dietary intervention: (1) basal rodent diet D12450H (fat 10% kcal, protein 20% kcal, carbohydrate 70% kcal) (Shenzhen) for the control group; (2) high-fat diet D12450 (fat 45% kcal, protein 20% kcal, carbohydrate 35% kcal) for the HF group; (3) high-fat diet D12450 supplemented with 20% w/w RS2 (high amylose maize starch with 56% RS2, Ingredion, Shanghai) for the HFRS group. Mice assigned to each treatment group were housed in two cages (two or four mice per cage). Body weight was recorded every two weeks. Food intake was recorded every three days and calculated monthly. Food efficiency ratio (FER) was calculated (weight gain divided by the weight of consumed feed) indicating efficiency of food intake. After 16 weeks of dietary intervention, mice feces and blood were collected prior to sacrifice by cervical dislocation.

Colon length was measured, and contents of cecum and colon were collected for measurement of SCFA production. Liver and colon tissues were harvested for histological evaluation and mRNA quantification. All the tissue samples were kept at -80°C before further analysis except from samples utilized for histological evaluation which were fixed in formalin. All procedures were conducted in compliance with institutional guidelines and were approved by the Animal Ethical Committee of Zhejiang University prior to initiating the study.

### Histological analysis

In order to evaluate possible dietary effects on organ, we preformed histologic evaluation on the liver and the colon specimens at the end of the study. Mice liver and proximal colon tissue samples were washed by sterile phosphate-buffered saline (PBS), fixed in 10% v/v formalin/PBS for 24h at room temperature, embedded in paraffin, sliced into 5 *μm* sections and processed with hematoxylin and eosin (H&E) stains. Colon histological scoring was determined by inflammatory cell infiltration (0-3) and tissue injury (0-3) based on previous description [46]. Non-alcoholic fatty liver disease (NAFLD) activity score (NAS) was calculated by evaluating the presence of steatosis (0-3), lobular inflammation (0-2), hepatocellular ballooning (0-2) and fibrosis (0-2) modified per a previous study [47]. Slides were examined under a microscope (Nikon Eclipse BO, Japan) at a magnification of x200 (colon) or x400 (liver) by an independent investigator blinded to the group assignments.

### Serum and fecal LPS levels measurement

Whole blood was allowed to clot at room temperature for 30 minutes and then centrifuged at 2,000 x g for 10 minutes in a refrigerated centrifuge. Serum was collected from the supernatant. 50mg stool were suspended in 0.5ml PBS, and supernatant was collected after centrifuging at 2000 rpm for 20 minutes. All the serums and stool supernatant were stored at -80°C prior to lipopolysaccharide (LPS) testing. LPS levels in serum and stool were measured by an Elisa kit (ColorfulGene Biological Technology Co. LTD, Wuhan, China).

### Mucin and inflammation cytokine mRNA detection

Expression of mucin 2 (MUC2) in the colon and inflammatory cytokines (tumor necrosis factor (TNF)-*α* interleukin (IL)-1*β*, IL-4 and IL-2) in the colon and liver were evaluated by measuring mRNA levels. Total RNA was isolated from the colon and liver tissues through Trizol (Ambion, USA) method and processed with a PrimeScript RT reagent Kit (Takara, Japan) to synthesize cDNA. Quantitative real-time PCR was performed in triplicate for each sample with Lightcycler 480 instrument (Roche Applied Science, Penzberg, Germany) using SYBR Premix Ex TaqTM II (Takara, Japan). *β*-actin gene was used as reference, and primer sequences used are listed in Table 1. Relative gene expression was expressed by fold change (2-ΔΔCt) relative to the expression in the control samples.

**Table 1:** Primer sequences.

**Gene**	**Forward primer (5'-3')**	**Reverse primer (5'-3')**
β-actin	GCAGGAGTACGATGAGTCCG	ACGCAGCTCAGTAACAGTCC
Muc2	GAAGCCAGATCCCGAAACCA	GAATCGGTAGACATCGCCGT
TNF-α	TAGCCAGGAGGGAGAACAGA	TTTTCTGGAGGGAGATGTGG
IL-1β	TTGACGGACCCCAAAAGATG	AGAAGGTGCTCATGTCCTCA
IL-4	GGTCTCAACCCCCAGCTAGT	GCCGATGATCTCTCTCAAGTGAT
IL-2	TGAGCAGGATGGAGAATTACAGG	GTCCAAGTTCATCTTCTAGGCAC

### PAS-AB and immunofluorescence staining for Mucin 2 protein

Paraffine-embedded colon tissue sections were serially deparaffinized in xylene and rehydrated in graded ethanol to distilled water. Periodic acid-Schiff-alcian blue (PAS-AB) staining was used to identify the negatively charged mucin expression in the colon. Following dewaxing and hydrating, antigen retrieval was performed in a microwave oven by incubating the sections in 10mM sodium citrate (pH 6). After naturally cooling, sections were washed 5 mins using PBS (pH 7.4) three times. Sections were blocked for 30 mins at room temperature in bovine serum albumin (BSA) to prevent nonspecific staining and incubated with primary antibody polyclonal rabbit anti-MUC2 antibody (1:800, Servicebio, GB 11344, Wuhan, China) overnight at 4°C. The primary antibody was recognized by incubating sections with secondary primary Alexa 488-labelled goat anti-rabbit IgG (1:400, Servicebio, GB25303, Wuhan, China) without light exposure for 50 mins. Sections were counterstained with 4’,6-diamidino-2-phenylindole (DAPI) and secured on antifade mountant.

PAS-AB staining cells in each crypt were counted in five different areas per section for each mouse in the three groups using a light microscope (400 x magnification, Nikon Eclipse BO, Japan). All fluorescent images were recorded by an upright fluorescent microscope (200 x magnification, Nikon Eclipse C1, Japan). Quantitative analysis of fluorescent stains was performed with Image J software by calculating the mean fluorescence intensity in each crypt in five regions per section for each mouse in the three groups. All the image analyses were conducted by two independent observers.

### Fecal DNA Extraction, PCR amplification, sequencing and analysis

Fecal microbial DNA extractions were performed by using TIANamp Stool DNA Kit (TIANGEN BIOTECH, cat. #DP328-02, Beijing, China). DNA concentration and quality were examined by Nanodrop 2000 UV-vis spectrophotometer (Thermo Scientific, Wilmington, USA) and 1% agarose gel electrophoresis, respectively. PCR amplification of bacterial 16s rRNA gene V3-V4 region was conducted by using barcoded primers 338F 5′-ACTCCTACGGGAGGCAGCAG -3′ and 806R 5′-GGACTACHVGGGTWTCTAAT-3′ by thermocycler PCR system (GeneAmp9700, ABI, USA). PCR reactions in triplicate with 20 *μ*l PCR mixtures, containing 4 *μ*l of 5 x FastPfu Buffer, 2 *μ*l of dNTPs (2.5mM), 0.8 *μ*l amplicon PCR forward (5 *μ*M) and reverse (5 *μ*M), 0.4 *μ*l of FastPfu Polymerase and 10 ng of microbial genomic DNA, were carried out using the following protocol: initial denaturation at 95 °C for 3 min, 27 cycles of denaturation at 95 °C for 30s, annealing at 55 °C for 30s, extension at 72 °C for 45s, and a final extension at 72 °C for 10 min. The amplicons were subsequently visualized on 2% agarose gels, purified by AxyPrep DNA Gel Extraction Kit (Axygen Biosciences, Union City, CA, USA), and the concentration was determined by using QuantiFluor™ -ST (Promega, USA).

Purified amplicons were mixed with appropriate proportion and paired-end sequenced (2 x 300) on an Illumina MiSeq platform (Illumina, San Diego, USA) according to standard protocol by Majorbio BioPharm Technology Co. Ltd. (Shanghai, China). Raw PE sequences were demultiplexed and quality-filtered by Trimmomatic and merged by FLASH (Fast Length Adjustment of Short Reads to Improve Genome Assemblies) as described previously [48]. Briefly, a 50-base pair (bp) sliding window was set and low-quality reads with score <20 were truncated from the 3′ end. Afterwards, paired-end reads with a minimum overlap length of 10 bp were assembled. No barcode mismatches were allowed, and primers were exactly matched allowing 2 nucleotide mismatching. The maximum acceptable reads mismatch rate was set at 20%. Reads containing ambiguous characters or could not be assembled were discarded. Optimized merged sequences were de-replicated, and single deletions were removed. Sequences with at least 97% similarity cutoff were clustered into operational taxonomic units (OTUs) using UPARSE (version 7.1 http://drive5.com/uparse/), and chimeric sequences were identified and removed by UCHIME. The taxonomy of 16S rRNA gene sequence was determined by Ribosomal Database Project (RDP) Classifier (version 2.2http://rdp.cme.msu.edu/) with the SILVA rRNA gene database (Release128 http://www.arb-silva.de) as reference using a confidence threshold of 70%. Data were analyzed using the software Quantitative Insights Into Microbial Ecology (QIIME) v 1.9.1 pipeline.

### Functional prediction

The 16S rRNA functional prediction was initially carried out by PICRUSt (Phylogenetic Investigation of Communities by Reconstruction of Unobserved States) to normalize OTUs. Normalized OTUs were categorized into Clusters of Orthologous Groups (COG). The descriptive and related functional information of each COG were parsed from the EggNOG (evolutionary genealogy of genes: Non-supervised Orthologous Groups, http://eggnog.embl.de/) database, and the abundance of each functional category was calculated based on the corresponding relation.

### Short chain fatty acid analysis

Colon (cecum and colon distal to the cecum) SCFA levels were analyzed using gas chromatography as previously described [49]. Briefly, 0.5 ml 10% (w/v) suspensions of colonic contents mixed with 0.1 ml 25% (w/v) metaphosphoric acid were stored at –20 °C for 24 hours, centrifuged at 14, 000 xg for 20 min, and filtered by 0.22 *μ*m membrane filters. SCFAs including acetic, propionic, butyric (n-butyric), valeric, isobutyric (i-butyric), and isovaleric (i-valeric) acids were separated using Shimadzu (GC-2010 Plus, Japan) with InterCap FFAP columns (0.25 mm × 30 mm × 0.25 mm). Peaks were integrated with GC Solution software, and SCFA content was quantified by a single-point internal standard method by using crotonic acid as an internal standard.

### Statistical analysis

Data were presented as mean ± standard error (SE) or median with quartiles according to their distributions. Differences in weight gain, food efficiency ratio, health score, histological score, colon length, LPS levels, mRNA relative expression levels, and intestinal SCFA production of the three groups were analyzed by one-way analysis of variance (ANOVA) with Tukey’s multiple comparison posttests for data with normal distribution or Kruskal-Wallis test with Dunn’s multiple comparisons posttest for data with non-normal distribution.

Mothur (v.1.30.1) was used to calculate within-community diversity (*α*-diversity: Sobs and Chao1 index reflect community richness; Shannon index represents community diversity) and group diversity indexes were compared by ANOVA with Tukey’s multiple comparison posttests. Differences in microbial communities of phylum, family, and genus levels, as well as, the functional prediction results among the three groups were further analyzed using Kruskal-Wallis test. All statistical tests were two-tailed, and P-values <0.05 were considered statistically significant. Analysis and figures were performed using IBM SPSS Statistics V22.0 software and GraphPad Prism v7.
